# Effects of aripiprazole long-acting two-injection start in patients diagnosed with schizophrenia in Huelva (Spain)

**DOI:** 10.1192/j.eurpsy.2023.2215

**Published:** 2023-07-19

**Authors:** A. Moleon, L. Duque, M. Martin-Bejarano

**Affiliations:** 1 Hospital Universitario Virgen del Rocio; 2 Instituto Andaluz de Salud Cerebral, Sevilla; 3 Hospital Universitario Juan Ramón Jimenez, Huelva; 4 Hospital Universitario 12 de Octubre, Madrid, Spain

## Abstract

**Introduction:**

Improving outcomes in schizophrenia generally involves an improvement in drug adherence. Aripiprazole two-injection start (TIS) is the newest option of available on the market, with limited data on its effects.

**Objectives:**

It was our goal to evaluate whether TIS has an effect on hospitalization rates, persistence and adverse events in patients with schizophrenia.

**Methods:**

This 12-months cross-sectional study included 32 patients suffering from schizophrenia (mean age 33.6 years; 22 males). We collected sociodemographic data on all individuals, hospitalization rates, persistence, use of neuroleptic drugs as well as adverse events.

**Results:**

Before starting TIS, the mean in terms of number of hospitalizations was 5.6, emergency department visits 8.7 and hospitalization days 12. After TIS, hospitalization rates was 22%, persistence 81%, adverse events were present in 3% of the patients and only 9% needed concomitant treatment with neuroleptic drugs.

**Conclusions:**

The findings imply that TIS should be considered a first-line treatment choice for schizophrenic patients. It results in a decrease in the use of hospital services, which might ease the socioeconomic healthcare burden.

**Disclosure of Interest:**

None Declared

